# Drug repurposing as add-on treatment strategy for mania and bipolar depression: systematic synthesis and qualitative appraisal of the existing meta-analytic evidence

**DOI:** 10.1192/j.eurpsy.2024.671

**Published:** 2024-08-27

**Authors:** D. Cavaleri, F. Bartoli, C. Crocamo, G. Carrà

**Affiliations:** ^1^Department of Medicine and Surgery, University of Milano-Bicocca , Monza, Italy; ^2^Division of Psychiatry, University College London, London, United Kingdom

## Abstract

**Introduction:**

In the complex pathophysiology of bipolar disorder (BD), increasing evidence supports the involvement of neurobiological abnormalities beyond the classical ones, suggesting them as potential alternative therapeutic targets. Several drugs approved for different indications have thus been repurposed for the treatment of BD, all of them supported by a plausible biological rationale. Some recent reviews have provided an update on these possible additional treatment options for mania and bipolar depression, but no systematic synthesis and qualitative evaluation of meta-analytic findings has been made.

**Objectives:**

To provide a guidance on the available evidence on these treatments and their potential role in clinical practice, we conducted an umbrella review of meta-analyses of randomized placebo-controlled trials investigating drugs repurposed as add-on treatments for mania and bipolar depression.

**Methods:**

We performed a systematic search and screening of the existing literature looking for the most up-to-date or comprehensive meta-analyses of randomized controlled trials (RCTs) on adults suffering from BD during an acute mood episode (mania or depression) which compared a repurposed drug and placebo as adjunctive treatments. We performed a critical appraisal according to “A MeaSurement Tool to Assess systematic Reviews” Version 2 (AMSTAR 2). We synthesized meta-analytic findings regarding efficacy, tolerability, and safety, also assessing the quality of evidence using the “Grading of Recommendations, Assessment, Development and Evaluations” (GRADE) approach.

**Results:**

In nine eligible meta-analyses investigating 12 drugs (four for mania and eight for bipolar depression) we observed a heterogeneous quality of reporting was according to AMSTAR 2.

In mania, allopurinol (for symptoms reduction and remission at 4-8 weeks) and tamoxifen (for response and symptoms reduction at 4-6 weeks) showed higher efficacy than placebo, with evidence of low and very low quality, respectively.

In bipolar depression, modafinil/armodafinil (for response, remission, and symptoms reduction at 6-8 weeks) and pramipexole (for response and symptoms reductionat 6 weeks) were superior to placebo, with low-quality evidence. Results on celecoxib and N-acetylcysteine were of low quality and limited to certain outcomes.

**Conclusions:**

Overall, the lack of evidence of high and moderate quality does not allow firm conclusions on the clinical utility of repurposed drugs as adjunctive treatments for mania and bipolar depression, limiting recommendations for their use in clinical practice. However, since some lines of evidence seem to hold some potential, and standard treatments for mania and bipolar depression remain not entirely satisfactory, the search for novel therapeutic targets and strategies for the management of BD warrants further research in the field.

**Disclosure of Interest:**

None Declared

